# Automated deep learning model for predicting pathological complete response in rectal cancer: A tool to organ-preserving strategies

**DOI:** 10.1007/s00384-026-05155-1

**Published:** 2026-05-22

**Authors:** Martin-Arevalo J., Lopez-Mozos F., Moro-Valdezate D., Perez-Santiago L., Palomo-Lopez I., Garcia-Botello S. A., Tarazona-Llavero N., Cabrera-Perez B., Riera-Cardona M., Lillo-Albert G., Millan M, Pla-Marti V.

**Affiliations:** 1 University of Valencia, Valencia, Spain; 2 Departament of General and Digestive Surgery, Department of Colorectal Surgery, Clinic Universitary Hospital of Valencia, Av. Blasco Ibáñez, 17. 46010 Valencia, Spain; 3 INCLIVA Biomedical Research Institute, Valencia, Spain; 4 Department of Medical Oncology, INCLIVA Biomedical Research Institute, University of Valencia, Valencia, Spain; 5 CIBERONC, Carlos III Health Institute, Madrid, Spain; 6 Division of Hematology/Oncology, Mass General Brigham Cancer Center, Harvard Medical School, Boston, MA USA; 7 Radiology Department, Clinic Universitary Hospital, Valencia, Spain

**Keywords:** Convolutional net, Rectal cancer, Neoadjuvant chemoradiotherapy, Pathological complete response, Deep learning, MRI, Watch-and-wait

## Abstract

**Background:**

Accurate identification of pathological complete response (pCR) after neoadjuvant chemoradiotherapy (nCRT) in locally advanced rectal cancer (LARC) remains a key clinical challenge. Clinical complete response is an imperfect surrogate, and pCR can only be definitively established after surgery. We developed a fully automated, segmentation-free deep learning model to support post-treatment response assessment using routine T2-weighted MRI.

**Methods:**

A longitudinal three-dimensional (3D) siamese convolutional neural network was trained using paired pre- and post-nCRT axial T2-weighted MRI volumes and a normalized signed voxel-wise difference map. The multitask framework simultaneously predicted rectal wall response (good response: modified Ryan score 0–1 vs poor response ≥ 2) and nodal status (ypN0 vs ypN +), from which pCR probability (ypT0N0) was derived. A retrospective single-center cohort of 195 patients was divided into training and independent test sets stratified by pCR status. Performance was evaluated using AUC-ROC and standard classification metrics with bootstrap-derived 95% confidence intervals.

**Results:**

In the independent test set (*n* = 49; pCR prevalence 18.5%), the model achieved an AUC-ROC of 0.71 (95% CI: 0.55–0.85) for pCR prediction. At the selected operating threshold, sensitivity was 100% (95% CI: 70.1–100) and negative predictive value (NPV) was 100% (95% CI: 81.6–100), with a specificity of 42.5% (95% CI: 28.5–57.8). The high NPV reflects the low prevalence of pCR in the study cohort and may vary across external populations.

**Conclusions:**

This fully automated longitudinal deep learning model demonstrated moderate discrimination and a high-sensitivity profile for pCR detection. Its performance suggests potential utility as a screening or triage tool to support multidisciplinary assessment, rather than to directly guide organ-preserving strategies. External multicenter validation is required before clinical implementation.

## Introduction

Locally advanced rectal cancer (LARC) represents a substantial proportion of newly diagnosed rectal cancers [1, 2], with neoadjuvant chemoradiotherapy (nCRT) followed by total mesorectal excision (TME) representing the standard of care for patients with stage II–III disease [3, 4]. This multimodality approach achieves pathological complete response (pCR) in approximately 15–25% of cases, defined as the absence of residual viable tumor cells in the resected specimen (ypT0N0) [5]. Achieving pCR is strongly associated with excellent oncological outcomes, including reduced local recurrence and improved disease-free survival, and has driven increasing interest in organ-preserving strategies such as watch-and-wait (W&W) in carefully selected patients who achieve a clinical complete response after nCRT [6, 7].

Accurate preoperative identification of pCR is therefore a critical clinical challenge, directly influencing patient selection for W&W and the avoidance of unnecessary radical surgery. Total mesorectal excision is associated with substantial morbidity, including bowel, urinary, and sexual dysfunction, as well as permanent stoma formation in a proportion of patients, significantly impairing quality of life [8]. However, differentiation between fibrosis and residual viable tumor remains challenging, particularly in the mesorectum and nodal compartments. This limitation contributes to considerable interobserver variability and suboptimal diagnostic accuracy. Meta-analyses have reported pooled sensitivities as low as 32% for mrTRG 1 in predicting pCR, highlighting the persistent risk of under-detection and consequent overtreatment of potential W&W candidates [1, 9].

In this context, artificial intelligence (AI) and deep learning (DL) have emerged as promising approaches to improve objective and reproducible prediction of treatment response from MRI. Several recent studies have demonstrated encouraging performance, particularly using T2-weighted and diffusion-weighted imaging, often outperforming conventional radiomics models [10–12]. For instance, recent approaches have integrated multiparametric MRI radiomics to assess fine tumor features, although they often require complex multi-sequence acquisition and expert-led feature extraction [13]. However, as highlighted in recent systematic reviews, most published approaches still depend on manual or semi-automatic tumor segmentation [14], introducing observer bias, increasing workflow complexity, and limiting scalability in routine clinical practice. Furthermore, relatively few models explicitly leverage longitudinal pre- and post-treatment information in a fully automated manner, and external validation remains scarce, hampering clinical translation [10, 12].

To address these limitations, there is a need for diagnostic tools that can analyze full-volume imaging data without the constraints of manual delineation. The primary aim of this study was to develop and internally validate a fully automated, segmentation-free 3D deep learning model based on longitudinal, whole-pelvis, axial T2-weighted MRI for the prediction of pCR in patients with LARC. By adopting a multitask learning strategy to simultaneously predict rectal wall response and nodal status, we sought to provide a clinically applicable decision-support tool that captures the complexity of post-treatment assessment in a fully objective and reproducible manner.

## Materials and methods

### Study design and patient cohort

This was a retrospective, single-center observational study conducted at a tertiary university hospital in Spain. The study protocol was approved by the institutional review board. The study adhered to STROBE guidelines for observational studies and TRIPOD-AI recommendations for prediction model development and validation [15, 16].

Consecutive patients with histologically confirmed locally advanced rectal adenocarcinoma (clinical stage II–III) who underwent nCRT, restaging MRI, and total mesorectal excision (TME) between January 2018 and December 2024 were screened for eligibility. Tumor staging was determined using baseline pelvic MRI and thoracoabdominal CT according to contemporary guidelines.

Exclusion criteria included non-adenocarcinoma histology, incomplete neoadjuvant treatment, absence of either pre- or post-nCRT axial T2-weighted MRI sequences, poor image quality precluding analysis, or incomplete pathological data.

Baseline MRI was obtained before initiation of neoadjuvant therapy, and restaging MRI was performed 6–10 weeks after completion of chemoradiotherapy, prior to surgery.

Post-neoadjuvant tumor regression in the rectal wall was evaluated using the modified Ryan tumor regression grading scale [17]. The primary pathological outcome was pathological complete response (pCR), defined strictly as the absence of any viable tumor cells in the surgical specimen (modified Ryan score 0, ypT0N0) [17]. All other surgical specimens showing any degree of residual viable tumor (modified Ryan scores 1, 2, or 3) were classified as non-pCR, regardless of the extent of regression.

Pre- and post-nCRT MRI examinations were performed using 1.5-T or 3-T scanners following standardized rectal cancer imaging protocols. To account for potential signal intensity heterogeneity arising from different field strengths and manufacturers, a rigorous preprocessing pipeline was applied, including N4 bias field correction and Z-score intensity normalization, ensuring consistent input features for the deep learning model. High-resolution axial T2-weighted fast spin-echo sequences were used for model input.

While diffusion-weighted imaging (DWI) is commonly used in rectal cancer restaging, it was excluded from this study to ensure cohort homogeneity. Due to evolving technical protocols, DWI was available in less than 20% of the total cohort, precluding its inclusion without significant selection bias.

All axial T2-weighted images were reconstructed into three-dimensional volumes and resampled to a standardized isotropic voxel spacing and fixed input dimensions. Pre- and post-nCRT volumes were jointly normalized to minimize inter-scan variability. A voxel-wise signed difference map (post − pre) was then computed to explicitly capture longitudinal treatment-induced changes. No manual or automated tumor segmentation was performed at any stage, and the model was trained using full unsegmented pelvic volumes.

### Deep learning model

The deep learning framework was based on a 3D Siamese Convolutional Neural Network backbone designed for longitudinal analysis. Input volumes consisted of pre- and post-nCRT axial T2-weighted images, resampled to isotropic voxel spacing and resized to a fixed dimension of 42 × 128 × 128 pixels. To enhance robustness and prevent overfitting, online data augmentation was performed using random rotations, flips, and scaling.

The model was trained using the Adam optimizer with an initial learning rate of 3 × 10 − 5 and a 'ReduceLROnPlateau' scheduler (factor 0.5, patience 10 epochs). To address the class imbalance (18.5% pCR), we employed a sigmoid focal cross-entropy loss function. Early stopping with restoration of the best-performing weights was implemented with a patience of 30 epochs. The final architecture utilized a multitask learning strategy to simultaneously predict rectal wall response and nodal status, from which the global pCR probability was subsequently derived.

The multitask model generated independent probability estimates for rectal wall response (ypT) and nodal status (ypN). The probability of pathological complete response (pCR) was subsequently derived as the joint probability of ypT0 and ypN0.

### Training, validation, and statistical analysis

The dataset was partitioned into a training set and an independent test set, stratified by pCR status. To ensure the robustness of the performance estimates and account for the limited number of events in the test cohort, non-parametric bootstrapping (1,000 iterations) was performed exclusively on the independent test set to derive 95% confidence intervals for all reported metrics (AUC, sensitivity, specificity, and NPV). Internal validation during the training phase was managed via a 20% validation split and early stopping to prevent overfitting.

Model performance was evaluated exclusively on the independent test set. Discriminative ability was assessed using receiver operating characteristic (ROC) curves and the area under the ROC curve (AUC). Sensitivity, specificity, positive predictive value (PPV), and negative predictive value (NPV) were calculated. Optimal probability thresholds were determined using Youden’s index. Precision–recall curves and confusion matrices were generated to further characterize performance under class imbalance.

To mitigate optimism bias due to the limited sample size, internal validation was performed using bootstrap resampling with 1,000 iterations. Performance metrics are reported as mean values with 95% confidence intervals derived from the bootstrap distributions.

To evaluate the reliability of the model's probabilistic outputs, we calculated the Brier Score and generated a Calibration Curve (Reliability Plot) using a quantile-based strategy. The Brier Score assesses the mean squared difference between predicted probabilities and actual outcomes, where a value of 0.25 represents the non-informative threshold (random guessing).

Model interpretability was explored using Gradient-weighted Class Activation Mapping (Grad-CAM) to visualize image regions contributing most strongly to model predictions.

Descriptive statistics were used to summarize the main demographic and clinical characteristics of the study cohort. Categorical variables are presented as relative frequencies with absolute counts in parentheses, while continuous variables are reported as mean ± standard deviation or median (range), according to their distribution. Comparisons between categorical variables were performed using the chi-square test.

All analyses were performed using Python (TensorFlow, scikit-learn, matplotlib). Statistical analyses were primarily descriptive, and statistical significance was defined as a two-sided *p* value < 0.05.

## Results

### Patient cohort

**Fig. 1:**
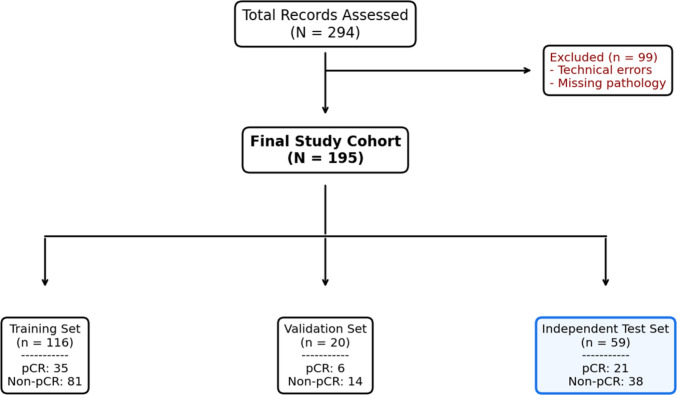
Study Flow Chart and Cohort Distribution. Process detailing the selection and exclusion of patients from the initial 294 records to the final study cohort (*N* = 195). Distribution into training (*n* = 116), validation (*n* = 20), and independent test sets (*n* = 59), including specific counts of actual pathological complete responses (pCR) for each group. *CSV, comma-separated values file; pCR, pathological complete response*

A total of 195 patients with locally advanced rectal adenocarcinoma fulfilled the inclusion criteria and were included in the final analysis. The dataset was split into a training set of 146 patients (75%), within which an internal validation subset was created using a 20% validation split during training, and an independent test set of 49 patients (25%), stratified by pCR status (Fig. 1).

The prevalence of pCR in the overall cohort was 18.5% (36/195), with 9 patients achieving pCR in the independent test set. Baseline demographic and clinical characteristics were generally well balanced between training/validation and test sets. Minor differences were observed in age and sex distribution, whereas key oncological variables (clinical stage, treatment regimen, and response patterns) were comparable across subsets (Table 1).

**Table 1:** Summary of main characteristics of study group. m: meters; kg: kilograms; ypN0: absence of metastatic lymph node involvement after neoadjuvant chemoradiotherapy; ypT0: absence of residual primary tumor infiltration after neoadjuvant treatment

	Train + Valid *n* = 146	Test *n* = 49	*p*-value
Age (years)	64.04 ± 11.21	70.86 ± 10.97	** < *0.001***
Gender (female)	49 (33.6%)	9 (18.4%)	***0.048***
BMI (Weight [kg]/height [m^2^])	25.27 (14.88–43.56)	25.15 (19.71–40.9)	0.557
Location of rectal cancer (Third)			0.127
Upper	14 (9.6%)	10 (20.4%)	
Middle	59(40.4%)	16 (32.7%)	
Lower	73 (50%)	23 (46.9%)	
Clinical Stage			0.212
II	19 (13%)	12 (24.4%)	
III	108 (74%)	29 (59.2%)	
IV	19 (13%)	8 (16.3%)	
Radiotherapy			0.390
Short course	66 (45.2%)	20 (40.8%)	
Long course	80 (54.8%)	29 (59.2%)	
Ryan Grade Regression			0.609
0	28 (19.2%)	11 (22.4%)	
1	19 (13%)	4 (8.2%)	
2	67 (45.9%)	20 (40.8%)	
3	32 (21.9%)	14 (28.6%)	
ypN0	103 (70.5%)	37 (75.5%)	0.584
Pathological Complete Response (ypT0 ypN0)	27 (18.5%)	9 (18.4%)	1.0

### Overall diagnostic pipeline

**Fig. 2:**
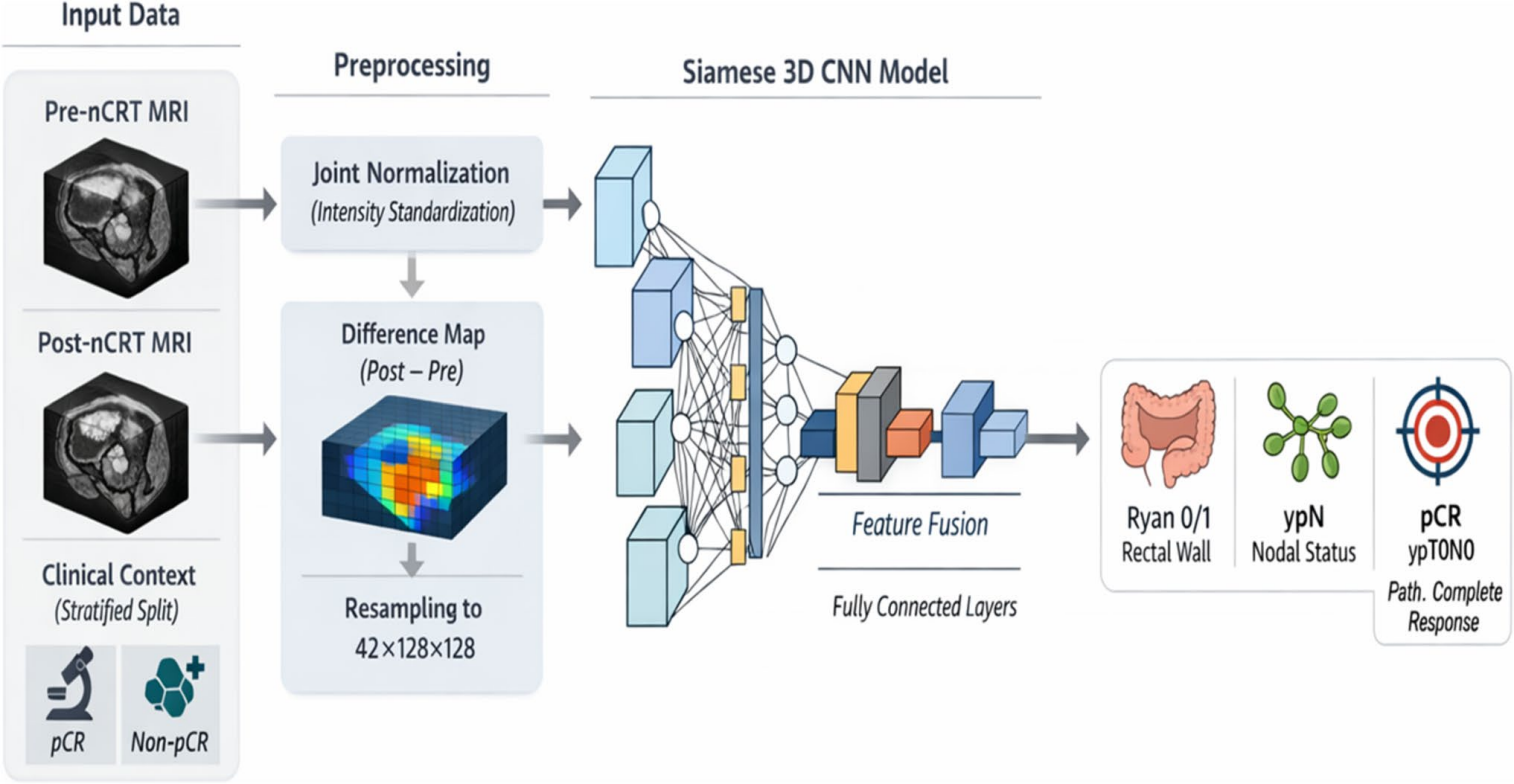
Study workflow and automated imaging pipeline. Schematic overview of the fully automated imaging pipeline for treatment response prediction. Pre- and post-nCRT axial T2-weighted MRI volumes were jointly normalized, and a signed voxel-wise difference map was computed. The three inputs (pre-treatment MRI, post-treatment MRI, and difference map) were processed by a 3D siamese convolutional neural network to generate three probabilistic outputs: rectal wall response (Ryan score 0/1), nodal status (ypN0 vs ypN +), and combined pathological complete response (pCR)

Figure 2 summarizes the overall diagnostic pipeline of the study. In brief, paired pre- and post-neoadjuvant T2-weighted MRI volumes were preprocessed, normalized, and used to generate a signed voxel-wise difference map. These three inputs (pre-nCRT MRI, post-nCRT MRI, and difference map) were then fed into a 3D siamese convolutional neural network trained in a multitask framework to predict rectal wall response, nodal status, and pCR. The pipeline was fully segmentation-free, relying exclusively on whole-volume imaging data and automated feature learning.

**Fig. 3:**
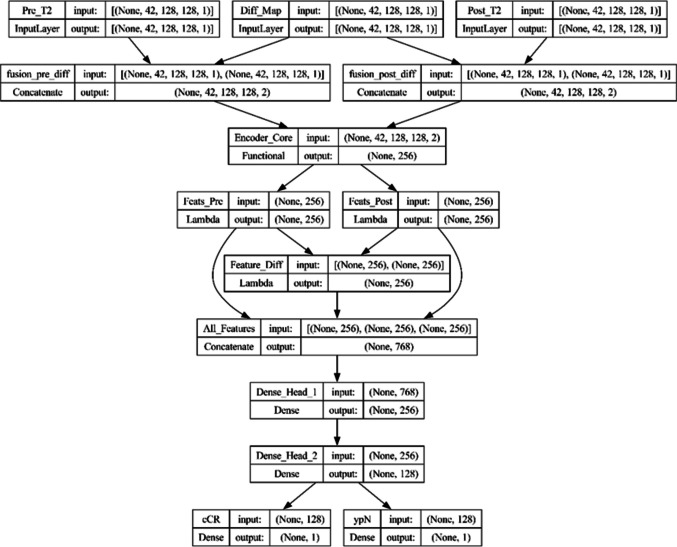
Architecture of the 3D siamese convolutional neural network. Two identical encoders with shared weights processed (**A**) pre-nCRT MRI fused with the difference map and (**B**) post-nCRT MRI fused with the difference map. Encoded representations were combined using absolute difference and concatenation, followed by fully connected layers and three independent sigmoid classification heads for rectal wall response, nodal status, and pCR prediction

The core of the system is a 3D siamese convolutional neural network architecture (Fig. 3), specifically designed to capture longitudinal changes between pre- and post-nCRT imaging while simultaneously learning shared representations for multiple clinically relevant outcomes.

### Model training and convergence

During training, the model demonstrated gradual and stable convergence without clear evidence of overfitting. The focal loss decreased progressively in both training and validation partitions and reached a plateau after approximately 25–35 epochs, after which learning rate reduction and early stopping were triggered.

**Fig. 4:**
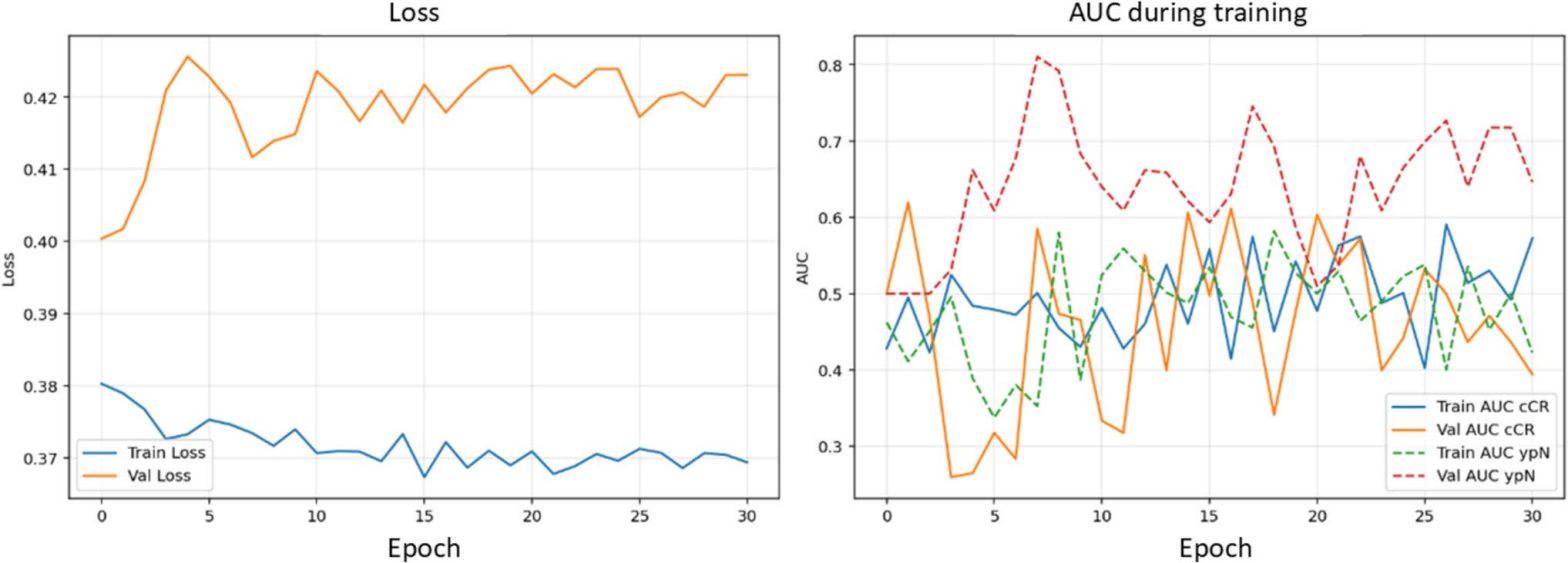
Training dynamics of the model. Evolution of (**A**) focal loss and (**B**) AUC during training and validation across epochs. The model showed gradual convergence without evidence of significant overfitting. Early stopping selected the epoch with the best validation performance

Training was conducted for a maximum of 150 epochs with early stopping (patience = 30) and ReduceLROnPlateau (factor = 0.5, patience = 10). The final selected model corresponded to the epoch with the best validation performance (Fig. 4).

**Fig. 5:**
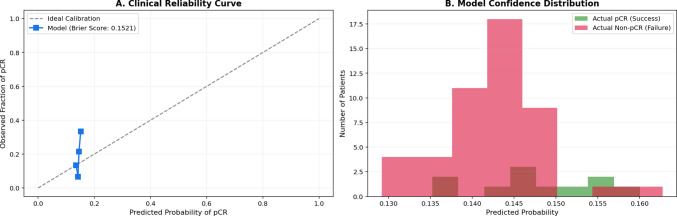
Model Calibration and Probability Reliability. (**A**) Reliability Curve: Calibration plot showing the agreement between the predicted probabilities of pathological complete response (pCR) and the observed outcomes on the independent test set. The model achieved a Brier Score of 0.152, significantly outperforming the non-informative threshold (0.25) and demonstrating strong alignment with the ideal calibration (dashed diagonal line). (**B**) Confidence Distribution: Histogram showing the distribution of predicted probabilities for patients with actual pCR (green) and non-pCR (red). The separation between groups and the concentration of cases in their respective probability deciles reflect the model's discriminative and probabilistic robustness

Regarding model calibration, the global pCR prediction achieved a Brier Score of 0.152 on the independent test set. The calibration curve (Fig. 5A) demonstrated strong alignment between predicted probabilities and observed pCR rates. The model showed a balanced distribution of confidence, with higher predicted probabilities effectively correlating with confirmed pathological complete responses (Fig. 5B).

### Predictive performance in the independent test set

Table 2 summarizes the main discrimination and classification metrics of the model for the three prediction tasks in the independent test cohort: rectal wall response, nodal status, and combined pCR.

**Table 2:** Summary of outcomes of model metrics

Metric	Ryan Score (Wall)	ypN (Nodes)	Combined pCR
AUC-ROC (95% CI)	0.713 (0.41–1.00)	0.680 (0.38–0.98)	0.708 (0.38–1.00)
AUC-PR/AP (95% CI)	0.425 (0.21–0.70)	0.469 (0.26–0.78)	0.294 (0.15–0.55)
Sensitivity (%)	54.5%	50.0%	100%
Specificity (%)	84.2%	91.9%	42.5%
NPV (%)	86.5%	85.0%	100%
Optimal Threshold	0.497	0.501	0.247

Internal validation using bootstrap resampling (1,000 iterations) demonstrated stable model performance across outcomes. The mean AUC for predicting pathological complete response (pCR) was 0.715 (95% CI: 0.555–0.857), for nodal status (ypN) 0.683 (95% CI: 0.476–0.879), and for pCR 0.703 (95% CI: 0.551–0.845).

To evaluate the model's clinical utility as a screening tool, an additional operating point was selected to maximize sensitivity (100%, 95% CI: 70.1–100), achieving a Negative Predictive Value of 100% and a specificity of 42.5% (95% CI: 28.5–57.8). These results indicate a conservative operating profile prioritizing the minimization of false-negative predictions.

### Prediction of rectal wall response

**Fig. 6:**
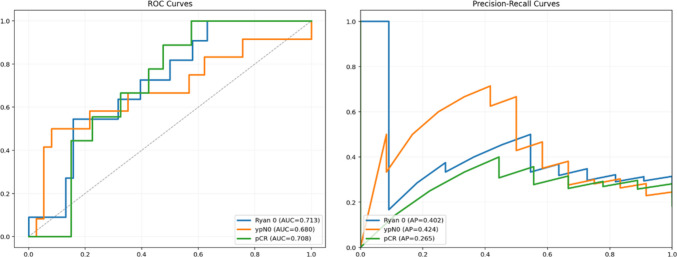
Model Discriminative Performance for pCR Prediction. (**A**) Receiver Operating Characteristic (ROC) Curve: The multitask Siamese network achieved an Area Under the Curve (AUC) of 0.71 (95% CI: 0.55–0.85) on the independent test set. The dashed diagonal line represents the performance of a random classifier. (**B**) Precision-Recall (PR) Curve: The model demonstrated an Area Under the PR Curve (AUC-PR) of 0.43. Given the 18.5% pCR prevalence in the cohort, the PR curve provides a more stringent assessment of the model's performance in the context of class imbalance. The shaded areas and error bars represent the 95% confidence intervals derived from 1,000 bootstrap iterations

For prediction of complete rectal wall response (modified Ryan score 0), the model achieved an AUC-ROC of 0.713 (95% CI: 0.409–1.000). At the Youden-optimized threshold (0.497), performance was characterized by higher specificity (84.2%) than sensitivity (54.5%), with a negative predictive value of 86.5%, indicating good ability to rule out residual mural disease. Precision–recall analysis yielded an AUC-PR of 0.425, reflecting moderate performance in the context of class imbalance (Fig. 6).

### Prediction of nodal status

For nodal status prediction (ypN0 vs ypN +), the model yielded an AUC-ROC of 0.680 (95% CI: 0.38–0.98). Using the optimal threshold of 0.501, specificity was high (91.9%), while sensitivity remained moderate (50.0%). The NPV was 85.0%, reflecting reasonable reliability for excluding nodal involvement, albeit with limitations inherent to MRI detection of micrometastatic disease. The AUC-PR for nodal prediction was 0.469, consistent with the moderate discriminative capacity observed in the ROC analysis (Fig. 6).

### Prediction of pathological complete response

For combined pCR prediction (ypT0N0), the model achieved an AUC-ROC of 0.708 (95% CI: 0.38–1.00). At the optimal threshold (0.247), the system correctly identified all patients with pCR, yielding a sensitivity of 100% (95% CI: 70.1–100.0%) and an NPV of 100% (95% CI: 81.6–100.0%).

Overall, discrimination assessed by AUC-ROC was highest for rectal wall response, whereas AUC-PR highlighted the greater challenge of accurate positive prediction for combined pCR in a low-prevalence cohort (Fig. 6).

### Model interpretability

**Fig. 7:**
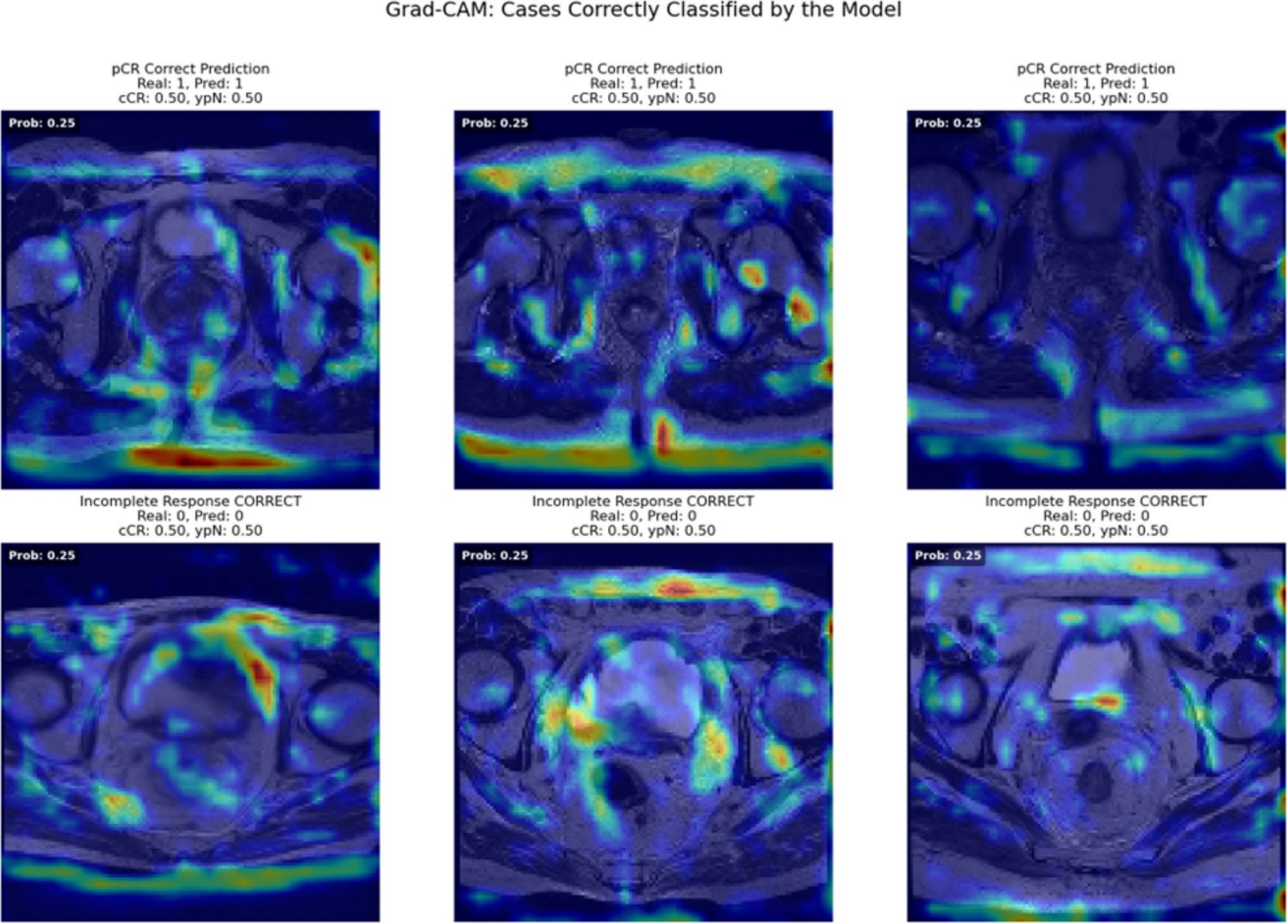
Grad-CAM visualization in representative test cases. Examples of Grad-CAM heatmaps overlaid on post-nCRT MRI and difference maps for (**A**) correctly predicted pCR, (**B**) correctly predicted non-pCR, (**C**) false positive, and (**D**) false negative cases. Activation maps highlight anatomically plausible regions, including the rectal wall, tumor bed, and mesorectal compartment

While Grad-CAM visualizations generally confirmed focus on the rectal wall and mesorectal fascia, identified failure modes included sporadic attention to adjacent non-target structures, such as small bowel loops or prominent vascular structures, particularly in cases with significant post-radiation inflammatory changes that altered the expected pelvic anatomy (Fig. 7).

## Discussion

In this study, we developed and internally validated a fully automated, segmentation-free longitudinal 3D deep learning model for predicting rectal wall response, nodal status, and pCR using paired pre- and post-nCRT T2-weighted MRI. In an independent test cohort, the model demonstrated moderate discrimination and a high-sensitivity profile for pCR detection.

By operating independently of patient age and sex, this model transcends traditional demographic constraints, establishing itself as a robust and unbiased biological proxy for predicting therapeutic outcomes in rectal cancer.

We utilized the Youden Index as the initial objective criterion for threshold selection to balance the overall diagnostic accuracy. However, in the clinical context of rectal cancer restaging, we prioritized a high-sensitivity operating point. We acknowledge that our NPV of 100% is influenced by the 18.5% pCR prevalence in our cohort. As predictive values are prevalence-dependent, this high NPV should be viewed as a performance characteristic specific to this study’s clinical setting. Future validation in cohorts with different prevalence rates is necessary to assess how these predictive values fluctuate in different clinical populations.

A key finding of our study is the high calibration of the multi-task Siamese network. While many deep learning models in radiomics suffer from overconfidence, our model’s Brier Score (0.152) indicates that the predicted probabilities are clinically actionable. The proximity of the reliability curve to the ideal diagonal suggests that the model can be used not just as a binary classifier, but as a risk-stratification tool. Specifically, the model’s conservative trend in high-probability ranges is particularly valuable in rectal cancer surgery, as it prioritizes safety by avoiding the overestimation of complete responses, thus minimizing the risk of under-treatment.

It is important to acknowledge that the definition of pCR is not fully standardized across the literature. While we adopted the strict criterion of ypT0N0, several studies in rectal cancer have defined pCR solely on the primary tumor bed (ypT0) irrespective of nodal status, and others have considered minimal residual disease (e.g., ypT1N0) as an acceptable response category [1, 10, 11]. These discrepancies substantially influence reported model performance, as broader definitions increase event rates and may simplify prediction tasks. Consequently, direct quantitative comparisons between studies should be interpreted with caution unless identical pathological endpoints are used.

Our framework stands out from previous radiomics and deep learning models by prioritizing clinical scalability through a segmentation-free and T2-only approach. While several studies [18, 19] and recent systematic reviews [1, 10, 11] often report higher pooled AUC values (0.85–0.91), these results frequently rely on labor-intensive manual segmentation or multimodal data (e.g., combining T2 with DWI or DCE-MRI). Our 3D Siamese CNN achieves a high clinical utility (NPV 100%) using only standard-of-care axial T2 sequences, bypassing the significant inter-observer variability and time constraints associated with manual contouring, which remain major barriers to clinical adoption. Furthermore, unlike models validated only on internal resampled cohorts, our use of an independent test set with bootstrap-derived confidence intervals provides a more rigorous and transparent estimate of performance, bridging the gap between experimental 'black-box' models and reliable clinical triage tools.

Unlike conventional delta-radiomics approaches, which rely on manual tumor segmentation and predefined radiomic features, our method analyzes longitudinal MRI changes directly in the image domain. By generating a normalized signed voxel-wise difference map and utilizing an end-to-end 3D siamese neural network, we eliminate the need for laborious ROI delineation. This is particularly relevant in the post-nCRT setting, where radiation-induced fibrosis and tissue remodeling make tumor boundaries increasingly difficult to define, even for mature segmentation tools.

While recent landmark studies [19], have achieved exceptional accuracy (AUC 0.99) using specialized sequences like diffusion kurtosis MRI, they often remain dependent on expert-led segmentation. Our approach prioritizes clinical scalability and minimizes interobserver variability by bypassing this bottleneck. Furthermore, by processing the entire volume, the model may capture complex spatial patterns and peritumoral changes that are often excluded in traditional segmentation-based models.

Although this strategy provides less explicit feature interpretability than hand-crafted radiomics, the use of Grad-CAM demonstrated that the model’s attention aligns with biological reality. In patients with pCR, activation maps corresponded primarily to areas of fibrosis and post-treatment remodeling, whereas in non-pCR cases, they highlighted regions suggestive of residual tumor. This supports the clinical and biological plausibility of the model’s predictions while offering a pragmatic, automated path for response assessment in standard clinical workflows.

The multitask design, simultaneously predicting rectal wall response and nodal status, reflects real-world decision-making in rectal cancer management, where both local and regional disease inform treatment strategy. The clinical importance of nodal status is underscored by recent efforts to apply the sentinel lymph node concept in rectal cancer [20], as well as the development of fluorescence-guided nodal harvesting techniques to improve staging accuracy [21]. Although MRI is inherently limited in detecting micrometastatic nodal disease, the shared representation learned by the model aligns with multidisciplinary reasoning rather than treating mural and nodal response as independent problems.

It is important to emphasize that the proposed 3D deep learning framework is designed to support, rather than replace, the Multidisciplinary Team (MDT) decision-making process. In clinical practice, our model could serve as a standardized 'second opinion' during restaging. By providing an objective probability of pCR based on the entire pelvic volume, it can help clinicians identify candidates for organ-preserving strategies who might otherwise be overlooked due to the high inter-observer variability of conventional MRI signs like fibrosis. Ultimately, the decision to proceed with a Watch & Wait protocol should remain a clinical one, integrating the model’s output with endoscopic findings, digital rectal examination, and the patient’s preferences.

Despite its promising results, this study has several limitations that must be addressed. First, it is a single-center retrospective analysis with a small test set, which is reflected in the wide confidence intervals of our metrics. Second, the model relies exclusively on T2-weighted sequences and lacks external validation in diverse cohorts. Furthermore, no prospective assessment was performed to evaluate its real-time clinical impact, and calibration reporting is limited by the sample size.

Several factors regarding the generalizability of our model must be acknowledged. Performance may be sensitive to technical variability, including different MRI scanner vendors and T2-sequence parameters. Furthermore, clinical heterogeneity—such as the use of Total Neoadjuvant Therapy (TNT) versus classic nCRT, variability in the interval-to-surgery, and shifts in pCR prevalence across different centers—could impact the model’s calibration and predictive accuracy. Future external validation in diverse, large-scale cohorts is essential to ensure the robustness of the framework across different institutional protocols.

We acknowledge that the 95% confidence intervals for some performance metrics, particularly the AUC, are relatively wide and reach the upper limit of 1.00. This reflects the inherent statistical uncertainty associated with the limited number of pathological complete response (pCR) events in our test cohort. Consequently, these results should be interpreted as a preliminary proof-of-concept of the multitask 3D framework, and larger multicentric studies are required to provide more precise estimates of its diagnostic accuracy.

Clinical integration of this model is proposed as a triage tool and digital second reader within the multidisciplinary team (MDT). Given the model’s moderate specificity, its primary role is not to independently dictate non-operative management, but to act as a conservative filter. A 'low-risk' prediction would trigger an expert clinical review (dedicated radiology and endoscopy) to confirm pCR, while a 'high-risk' prediction could reinforce the decision for surgical intervention. This workflow ensures the model acts as a safety net, identifying candidates for organ preservation without replacing clinical judgment.

In conclusion, a longitudinal 3D siamese deep learning model based on paired pre- and post-nCRT T2-weighted MRI and a normalized signed difference map showed moderate discrimination for rectal wall response and nodal status, and high sensitivity and negative predictive value for pathological complete response in an independent test set.

## Data Availability

The datasets generated during and/or analyzed during the current study are not publicly available due to institutional data protection policies regarding clinical imaging but are available from the corresponding author on reasonable request.
